# Haemolysis and myocardial and neural injury after monopolar pulsed field ablation using a novel lattice-tip catheter to treat atrial fibrillation

**DOI:** 10.1093/europace/euaf210

**Published:** 2025-09-09

**Authors:** Christian Gold, Paul Pratz, Anastasia Falagkari, Victoria Johnson, Florian Post, Esther Roth, Jana Kupusovic, Julia W Erath-Honold, Dominik Linz, David Leistner, Reza Wakili, Laura Rottner

**Affiliations:** Department of Cardiology and Vascular Medicine, University Heart and Vascular Center Frankfurt, Goethe University Frankfurt, Theodor-Stern Kai 7, Frankfurt am Main 60590, Germany; Department of Cardiology and Vascular Medicine, University Heart and Vascular Center Frankfurt, Goethe University Frankfurt, Theodor-Stern Kai 7, Frankfurt am Main 60590, Germany; Department of Cardiology and Vascular Medicine, University Heart and Vascular Center Frankfurt, Goethe University Frankfurt, Theodor-Stern Kai 7, Frankfurt am Main 60590, Germany; Department of Cardiology and Vascular Medicine, University Heart and Vascular Center Frankfurt, Goethe University Frankfurt, Theodor-Stern Kai 7, Frankfurt am Main 60590, Germany; Department of Cardiology and Vascular Medicine, University Heart and Vascular Center Frankfurt, Goethe University Frankfurt, Theodor-Stern Kai 7, Frankfurt am Main 60590, Germany; Department of Cardiology and Vascular Medicine, University Heart and Vascular Center Frankfurt, Goethe University Frankfurt, Theodor-Stern Kai 7, Frankfurt am Main 60590, Germany; Department of Cardiology and Vascular Medicine, University Heart and Vascular Center Frankfurt, Goethe University Frankfurt, Theodor-Stern Kai 7, Frankfurt am Main 60590, Germany; Department of Cardiology and Vascular Medicine, University Heart and Vascular Center Frankfurt, Goethe University Frankfurt, Theodor-Stern Kai 7, Frankfurt am Main 60590, Germany; Department of Cardiology, Maastricht University Medical Center and Cardiovascular Research Institute Maastricht, Maastricht, The Netherlands; Department of Biomedical Sciences, Faculty of Health and Medical Sciences, University of Copenhagen, Copenhagen, Denmark; Department of Cardiology and Vascular Medicine, University Heart and Vascular Center Frankfurt, Goethe University Frankfurt, Theodor-Stern Kai 7, Frankfurt am Main 60590, Germany; Department of Cardiology and Vascular Medicine, University Heart and Vascular Center Frankfurt, Goethe University Frankfurt, Theodor-Stern Kai 7, Frankfurt am Main 60590, Germany; Department of Cardiology and Vascular Medicine, University Heart and Vascular Center Frankfurt, Goethe University Frankfurt, Theodor-Stern Kai 7, Frankfurt am Main 60590, Germany

**Keywords:** Atrial fibrillation, Pulsed field ablation, Haemolysis, Myocardial biomarkers, S100

## Abstract

**Aims:**

The aim of this study was to assess the risk of haemolysis and the extent of myocardial and neural injury after monopolar, biphasic pulsed field ablation (PFA) using a lattice-tip catheter in comparison to single-shot PF ablation platforms employing bipolar, biphasic waveforms.

**Methods and results:**

This prospective study included consecutive patients undergoing PFA for atrial fibrillation (AF) using the Affera™ mapping and ablation system (*n* = 40). Biomarkers for haemolysis (haptoglobin, lactate dehydrogenase, bilirubin), myocardial injury [high-sensitive troponin T, creatine kinase (CK), creatine kinase MB (CK-MB)], neurocardiac injury (S100), and renal function (creatinine) were assessed pre- and within 24 h post-ablation. A subgroup analysis of first-time pulmonary vein isolation-only procedures compared biomarker changes across Affera™, Farapulse™ (PFA-F), and PulseSelect™ (PFA-P). Post-procedural haemolysis occurred across all PFA platforms. The decrease in Δhaptoglobin was most pronounced in PFA-F [Affera^TM^: (−) 13.8 ± 18.5 vs. PFA-P: (−) 36.8 ± 35.9 vs. PFA-F: (−) 60.7 ± 26.3 mg/dL, *P* = <0.001], without haemolysis-related complications. Affera^TM^ shows a trend towards a higher increase in myocardial injury markers (Δtroponin, 1537 [580] vs. 970 [1023] vs. 1051 [592] pg/mL, *P* = 0.180; ΔCK, 232 [168] vs. 153 [132] vs. 102 [144] U/L, *P* = 0.006; ΔCK-MB, 28.5 [15.3] vs. 14.6 [12.4] vs. 13.6 [10.5] U/L, *P* = 0.055, for Affera ^TM^, PFA-P, and PFA-F, respectively). After ablation, S100 increased in PFA-P and PFA-F, but not in Affera^TM^.

**Conclusion:**

Post-procedural haemolysis after PFA for AF treatment is common and occurs across all PFA platforms. Pulsed field ablation using Affera^TM^ results in more myocardial injury than bipolar PFA systems with no indication of neural damage.

## Graphical Abstract

**Graphical Abstract:**
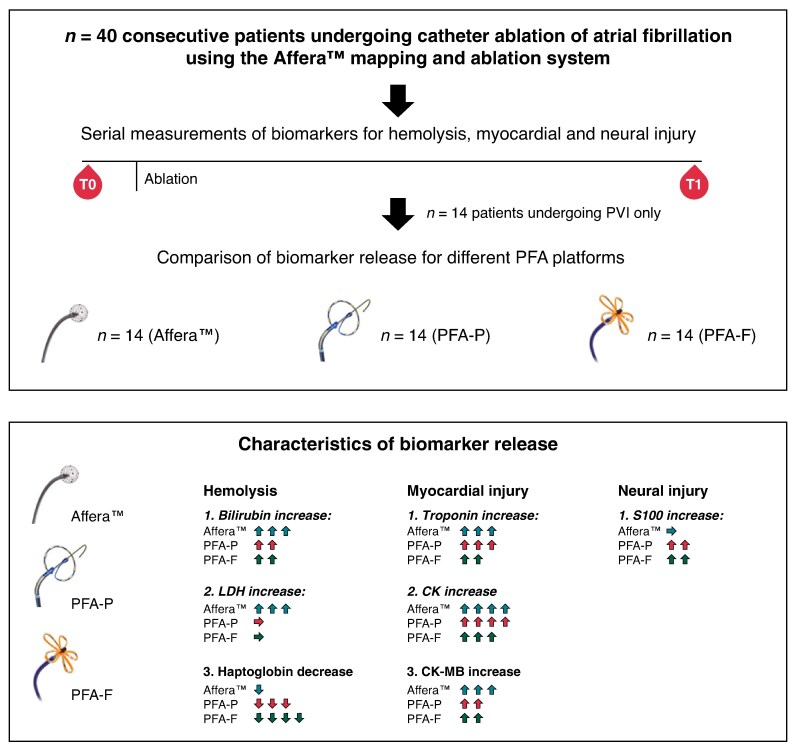

What’s new?

- Post-procedural haemolysis after pulsed field ablation (PFA) is common and occurs regardless of the PFA waveform, ablation platform, and catheter type.
- Pulsed field ablation using a monopolar, biphasic PF waveform and a lattice-tip catheter seems to be associated with a higher increase in myocardial injury markers when compared to bipolar, biphasic PFA waveform applied by single-shot catheters.
- No evidence of neural injury was observed after monopolar, biphasic PFA using a lattice-tip catheter, as indicated by unchanged S100 levels following ablation.

## Introduction

Pulsed field ablation (PFA) represents a novel energy source for catheter ablation of cardiac arrhythmias. It employs high-energy, ultrashort electrical pulses to permanently enhance the permeability of cell membranes (electroporation), preferentially affecting myocardial tissue. This method is unique in inducing non-thermal cell death, unlike radiofrequency (RF), cryoablation, or laser ablation, which rely on thermal destruction for lesion formation.^1,2^ In addition to its promising efficacy and clinical outcome profile, PFA—due to its cardioselectivity—seems to overcome typical complications of atrial fibrillation (AF) ablation (e.g. atrio-oesophageal fistula or phrenic nerve palsy).^3^ However, with increasing numbers of clinical cases performed, novel energy-specific complications are discovered. One example is haemolysis-induced renal failure, which has been described as a rare new complication in the largest global database of PFA, the MANIFEST 17K, which includes over 17 000 patients.^4^ Several previous studies, mainly describing haemolysis marker after single-shot bipolar PFA systems, assume that severe haemolysis with renal failure seems to occur only with extensive ablation and haemolysis in PFA.^4,5^ While current observations of haemolysis seem harmless, the potential for haemolytic effects associated with PFA remains not fully understood, and it is unknown whether the risk and extent of haemolysis following PFA vary by PFA waveform characteristics and catheter design.

Recently, a novel mapping and ablation platform (Affera^TM^, Medtronic Inc.) combined with a unique mapping and ablation catheter (Sphere-9^TM^, Medtronic Inc.) was introduced.^6,7^ The tip of this unique irrigated mapping and ablation catheter is lattice shaped with nine mini-electrodes on its surface and one central electrode. This catheter can apply monopolar biphasic PFA as well as RF according to anatomical locations and operator’s choice.^8^

This study sought to evaluate the risk of haemolysis and the extent of myocardial and neural injury after AF ablation procedures using Affera^TM^ and its novel monopolar, biphasic PFA lattice-tip catheter (Sphere-9^TM^) and to compare biomarker changes to two single-shot PFA systems using bipolar, biphasic PFA waveforms: the pentaspline PFA catheter (Farapulse^TM^) and the circular PFA catheter (PulseSelect^TM^).

## Methods

### Study population

Consecutive patients suffering from symptomatic AF, who underwent AF ablation using the novel Affera^TM^ mapping and ablation system in combination with the Sphere-9^TM^ catheter at the University Heart and Vascular Center Frankfurt between February 2025 and May 2025 were included into this prospective analysis. Blood collection was performed at two timepoints: (i) the day of the procedure and (ii) within 24 h after the procedure. Myocardial injury markers [creatine kinase (CK), creatine kinase MB (CK-MB), and high-sensitive troponin T], haemolysis markers [haptoglobin, lactate dehydrogenase (LDH), and bilirubin], and kidney function parameters (creatinine) as well as the release of neuronal biomolecules (S100) following PFA were analysed.

The Affera^TM^ cohort included *n* = 40 patients who received first-time pulmonary vein isolation (PVI), reablation procedures aiming for reisolation of previously targeted PVs, and also extended ablation beyond PVI. In order to compare the Affera^TM^ cohort to other established PFA platforms, blood samples of patients undergoing first-time ablation procedures only aiming for PVI with the Affera^TM^ system (*n* = 14) were compared to blood samples from patients undergoing first-time PVI procedures using either the pentaspline catheter (Farapulse^TM^, Boston Scientific; PFA-F, *n* = 14) or a circular PFA catheter (PulseSelect^TM^, Medtronic Inc.; PFA-P, *n* = 14) during the same time period.

All patients gave confirmed written consent. Data analysis and handling was performed in accordance with the Declaration of Helsinki. This analysis was approved by the institutional review board and the local ethics committee (Frankfurt Arrhythmia Registry, FRAME: registration number: 2023-1379).

### Periprocedural management

Prior to the procedure, transthoracic and transoesophageal echocardiography was performed to rule out intracardiac thrombi and to assess the left atrial diameter. No additional pre-procedural imaging was performed. Atrial fibrillation ablation was performed on uninterrupted oral vitamin K-anticoagulation with an INR of 2.0–3.0 on the day of the procedure. In patients treated with novel oral anticoagulants (NOACs), anticoagulation was paused on the day of the procedure.

Pericardial effusion after ablation procedures was ruled out in all patients by transthoracic echocardiography. Pre-existing therapy with NOACs was reinitiated 6 h after the ablation procedure. Anticoagulation was continued for at least 3 months and based on the individual CHA_2_DS_2_-VA score thereafter. Proton pump inhibitors were given for at least 6 weeks after ablation.

### Ablation procedures using the Affera^TM^ mapping and ablation system

Catheter ablation was performed under deep sedation using fentanyl and propofol. Vital parameters (pulse, blood pressure, and oxygen saturation) were continuously monitored. Heparin boluses were given targeting an activated clotting time > 300 s.

A diagnostic catheter was introduced via the right femoral vein and positioned within the coronary sinus. A single transseptal puncture was performed under fluoroscopic guidance using a modified Brockenbrough technique and an 8.5 F transseptal sheath (SL 0, Abbott Medical, Inc., St Paul, MN) was introduced into the left atrium (LA). Selective pulmonary vein (PV) angiography was performed to identify the PV ostia. As soon as the activated clotting time level was >300 s, the mapping and ablation catheter was introduced, and a 3D bipolar voltage map of the LA and the PVs was created.

The ostia of the ipsilateral PVs were tagged according to PV angiographies and local electrical information. For PVI or Re-PVI PF, energy was applied around the posterior aspect of the PV ostia and either PF or RF energy at the anterior aspect in order to isolate the PVs.

In patients with extensive LA low voltage areas or with additional atrial tachycardia, linear lesions were applied at operator’s discretion. If linear lesion sets at the posterior LA wall (roof line or posterior wall isolation) were performed, only PFA was used. Along the anterior wall and the mitral isthmus area, RF or PFA was applied at operator’s discretion. In patients with documentation of typical cavotricuspid isthmus (CTI)-dependent atrial flutter, the CTI was targeted using RF.

After completion of both ipsilateral circles around the PVs, another voltage map of the LA and the PVs was generated, and PV isolation was evaluated according to bipolar information and the absence of PV potentials on the lattice-tip catheter along the ablation line. If additional linear lesions were set during the procedures, bidirectional block was also confirmed by remapping as well as several stimulation manoeuvres. In case of catheter instability and requirement for more mechanical support, a steerable sheath (Agilis™, Abbott) was used at the discretion of the operator.

### Statistical analysis

Continuous variables were expressed as mean ± standard deviation (SD) in case of normal distribution or as median with interquartile range (IQR) in case of non-normal distribution. Categorical variables are reported as absolute numbers with corresponding percentages. The assumption of normal distribution was assessed using the Shapiro–Wilk test, and homogeneity of variances was tested with Levene’s test.

For paired samples in a two-group comparison, a paired *t*-test was performed in case of normal distribution, and the Wilcoxon signed-rank test was used for non-normally distributed data. For group comparison (>2 groups) of continuous variables, one-way ANOVA was used when normal distribution was present, applying Fisher’s ANOVA in case of equal variances and Welch’s ANOVA for unequal variances. Non-normally distributed data were analysed using the Kruskal–Wallis test. If overall significance was detected, pairwise *post hoc* comparisons were conducted based on distribution and variance characteristics: the Tukey test was applied for normally distributed and homoscedastic data, the Games–Howell test for normally distributed data with heteroscedasticity, and the Dwass–Steel–Critchlow–Fligner test for non-normally distributed data.

Categorical variables were compared using the χ^2^ test or Fisher’s exact test when cell counts were below 5. Correlation analysis was performed using Pearson’s correlation coefficient for normally distributed data and Spearman’s rank correlation for non-normally distributed data.

*P*-values of <0.05 were considered statistically significant. All analyses were performed with GraphPad Prism (Version 10.4.1).

## Results

### Baseline characteristics

A total of 68 patients (*n* = 40 Affera^TM^, *n* = 14 PFA-P, *n* = 14 PFA-F) suffering from symptomatic AF undergoing catheter ablation were included into our analysis. The median patient age was 65 [15] years with 25/40 (62.5%) male patients in the Affera™ group. In the PFA-P group, 10/14 patients (71.4%) were male with a median age of 65 [9] years; in the PFA-F group, 9/14 patients (64.3%) were male with a median age of 70 [11] years. Detailed patient baseline characteristics are depicted in *Table 1*.

**Table 1:** Baseline characteristics

Variable	Affera^TM^ (*n* = 40)	Affera^TM^ (*n* = 14)	PFA-P (*n* = 14)	PFA-F (*n* = 14)	*P*-value^a^
Gender, male (%)	25 (62.5)	8 (57.1)	10 (71.4)	9 (64.3)	0.919
Age, years, median (IQR)	67 (15)	70.1 (8.26)	65.1 (9.47)	70.4 (11.4)	0.292
BMI, kg/m², mean (SD)	29.5 (4.28)	30.7 (4.86)	28.7 (4.23)	26.2 (4.69)	0.048
Arterial hypertension, *n* (%)	26 (65)	9 (64.3)	9 (64.3)	12 (85.7)	0.406
Diabetes mellitus, *n* (%)	4 (10)	2 (14.3)	3 (21.4)	0 (0)	0.340
Coronary artery disease, *n* (%)	12 (30)	6 (42.9)	4 (28.6)	4 (28.6)	0.772
ICM, *n* (%)	4 (10)	2 (14.3)	2 (14.3)	1 (7.1)	1.000
CHAD2VA score, median (IQR)	2 (2.25)	2.64 (1.6)	2.43 (1.74)	3.21 (1.76)	0.458
LVEF, %, median (IQR)	55 (5)	55 (3.75)	55 (10)	60 (12.5)	0.365
LA area, cm², mean (SD)	26.1 (6.42)	22.9 (4.9)	28.1 (5.97)	27.2 (4.83)	0.082
NT-proBNP, pg/mL, median (IQR)	629 (819)	232 (807)	398 (1173)	618 (1248)	0.424
Paroxysmal AF, *n* (%)	19 (47.5)	10 (71.4)	9 (64.3)	10 (71.4)	1.000

BMI, body mass index; ICM, ischaemic cardiomyopathy; LVEF, left ventricular ejection fraction; LA, left atrium; AF, atrial fibrillation; PFA-P, PulseSelect; PFA-F, Farawave; SD, standard deviation; IQR, interquartile range.

^a^The reported *P*-values refer exclusively to the subgroup analysis and do not include the Affera^TM^ cohort (*n* = 40).

### Procedural data

In 14/40 (35%) of all Affera^TM^-guided procedures, PVI only was performed, whereas 26/40 (65%) patients underwent Re-PVI and/or ablation of additional linear lesions (5 roof lines, 12 posterior wall lesions, 9 anterior lines, and 9 CTI lines). All PVs were successfully isolated with a first-pass isolation rate of 100%. All linear lesion sets were finally bidirectionally blocked, and the posterior box was electrically isolated.

For the overall Affera^TM^ cohort, the median procedure time was 86 [27] min. The median fluoroscopy time and dose area product were 6 [6] min and 6.27 [6.91] Gycm^2^, respectively. Within the PVI-only group, the procedure time was significantly longer for the Affera^TM^ group when compared to procedures using the pentaspline or circular PFA catheter (94 ± 31.4 vs. 42.4 ± 12.1 vs. 43.7 ± 18 min, for Affera^TM^, PFA-P, and PFA-F, respectively, *P* = <0.001; *Table 2*). However, the fluoroscopy time (Affera^TM^, 6 [2.5]; PFA-P, 13.5 [4.05]; PFA-F, 13.3 [5.38] min, *P* = <0.001) and dose area product (Affera^TM^, 5.78 [3.45]; PFA-P, 15.9 [8.83]; PFA-F, 16.1 [7.99] Gy*cm², *P* = <0.001) were significantly lower for patients treated by Affera^TM^ using the lattice-tip catheter, when compared to pentaspline or circular PFA catheter guided (*Table 2*). Further detailed procedural data are given in *Table 2*.

**Table 2:** Procedural data

Variable	Affera^TM^ (*n* = 40)	Affera^TM^ (*n* = 14)	PFA-P (*n* = 14)	PFA-F (*n* = 14)	*P*-value^a^
Procedural time, min, median (IQR)	86 (27)	94 (31.4)	42.4 (12.1)	43.7 (18)	<0.001
Fluoroscopy time, min, median (IQR)	6 (6)	6 (2.5)	13.5 (4.05)	13.3 (5.38)	<0.001
Total radiation dose, Gy*cm², median (IQR)	6.27 (6.91)	5.78 (3.45)	15.9 (8.83)	16.1 (7.99)	0.007
PVI only, *n* (%)	14 (35)	14 (100)	14 (100)	14 (100)	1.000
Total number of applications, *n*, mean (SD)	66.6 (24.3)				
Total number of PFA applications, *n*, mean (SD)	59.5 (24.5)	62 (37)	32 (2.75)	32 (4.0)	0.009
Total number of applications in veins, *n*, mean (SD)	54.5 (27.4)				
Total number of PFA applications in veins, *n*, mean (SD)	52.5 (27.5)				

PVI, pulmonary vein isolation; PFA, pulsed-field ablation; PFA-P, PulseSelect; PFA-F, Farawave; SD, standard deviation; IQR, interquartile range.

^a^The reported *P*-values refer exclusively to the subgroup analysis and do not include the Affera^TM^ cohort (*n* = 40).

One major complication in the form of a pericardial tamponade, which was treated successfully with pericardiocentesis, occurred during AF ablation using the Affera^TM^ system. No further major or minor complications occurred.

### Post-procedural haemolysis, myocardial and neural injury

**Figure 1:**
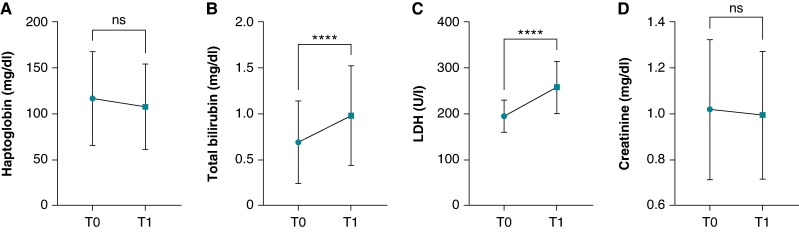
Temporal dynamics of (*A*) LDH (U/L), (*B*) total bilirubin (mg/dL), (*C*) haptoglobin (mg/dL) and (*D*) creatinine (mg/dL) from baseline (T0) compared to Day 1 (T1) post-PVI (Affera^TM^, *n* = 40). ns, *P* > 0.05; ****, *P* ≤ 0.0001.

Within the overall Affera^TM^ cohort (*n* = 40), venous blood samples before and on Day 1 after successful AF ablation showed a significant increase of haemolysis parameters LDH (194 ± 35 vs. 257 ± 57.2 U/L, *P* < 0.001) and bilirubin (0.69 ± 0.45 vs. 0.97 ± 0.54 mg/dL, *P* < 0.001) (*Figure 1*). A numerical, but no statistically significant, decrease was documented for haptoglobin on Day 1 compared to baseline (116 ± 51.1 to 107 ± 46.4 mg/dL, *P* = 0.153). Haemoglobin significantly decreased after ablation (13.2 [2.05] to 12.7 [1.25] mg/dL, *P* = 0.003). The number of PFA applications did not correlate with the decrease of Δhaptoglobin (*r* = 0.045; *P* = 0.803) and increase in Δbilirubin (*r* = 0.047; *P* = 0.792). A negative correlation was observed in ΔLDH and PFA applications [r = (−) 0.367; *P* = 0.031]. Renal function, assessed by creatinine levels, remained unchanged before and after the procedure (1.02 ± 0.31 vs. 0.99 ± 0.28 mg/dL, *P* = 0.332; *Figure 1*).

Myocardial injury markers CK (79 [50] vs. 214 [175] U/L, *P* = <0.001), CK-MB (16 [5] vs. 37 [23.5] U/L, *P*=<0.001) and troponin (9.4 [11.2] vs. 966.0 [1158] pg/mL, *P* = <0.001) significantly increased after ablation. No significant dynamic in S100 plasma concentration (0.056 [0.026] vs. 0.056 [0.034] μg/L, *P* = 0.762) was observed.

**Figure 2:**
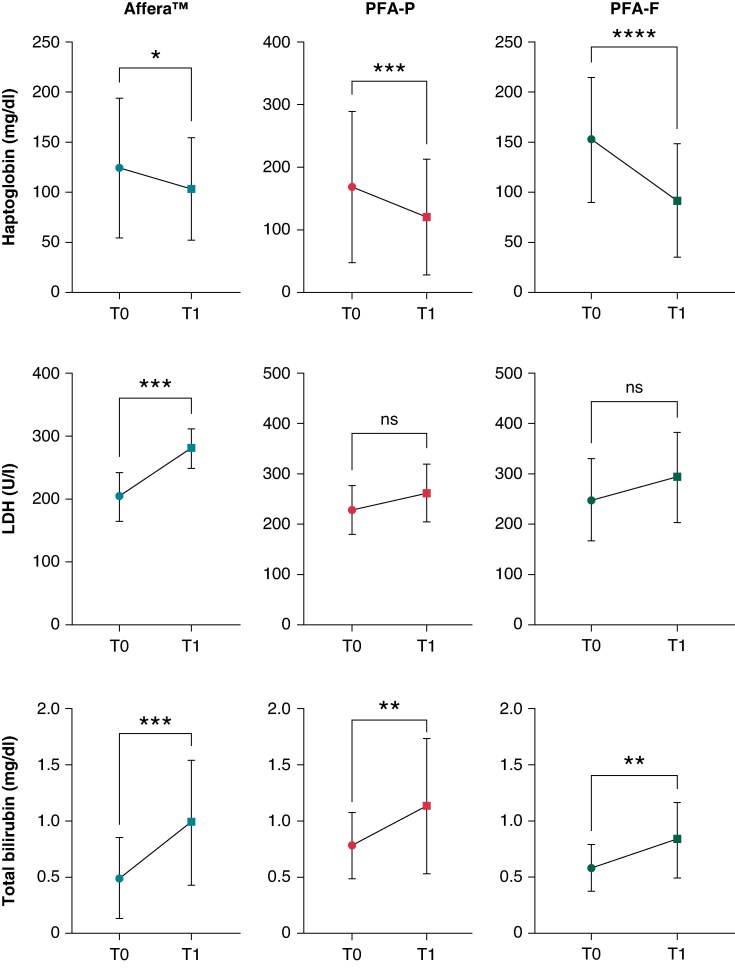
Temporal dynamics of haemolysis parameters, baseline (T0) compared to Day 1 (T1) post-PVI using different PFA platforms. Analysis of haemolysis parameters following PVI revealed a significant increase in bilirubin levels and a significant decrease in haptoglobin across all three PFA modalities. LDH levels increased significantly in the Affera^TM^ group, whereas no significant change was observed in PFA-P and PFA-F. ns, *P* > 0.05; *, *P* < 0.05; **, *P* < 0.01; ***, *P* < 0.001; ****, *P* ≤ 0.0001.

Within the PVI-only subcohorts (*n* = 14 Affera^TM^, *n* = 14 PFA-P, *n* = 14 PFA-F), parameters were obtained indicating haemolysis for all PFA platforms, as evidenced by a significant increase in bilirubin and decrease in haptoglobin on the day after ablation compared to baseline (*Figure 2*). Regarding LDH-level changes, a significant increase was only observed in Affera^TM^ but not in PFA-P and PFA-F patients.

**Figure 3:**
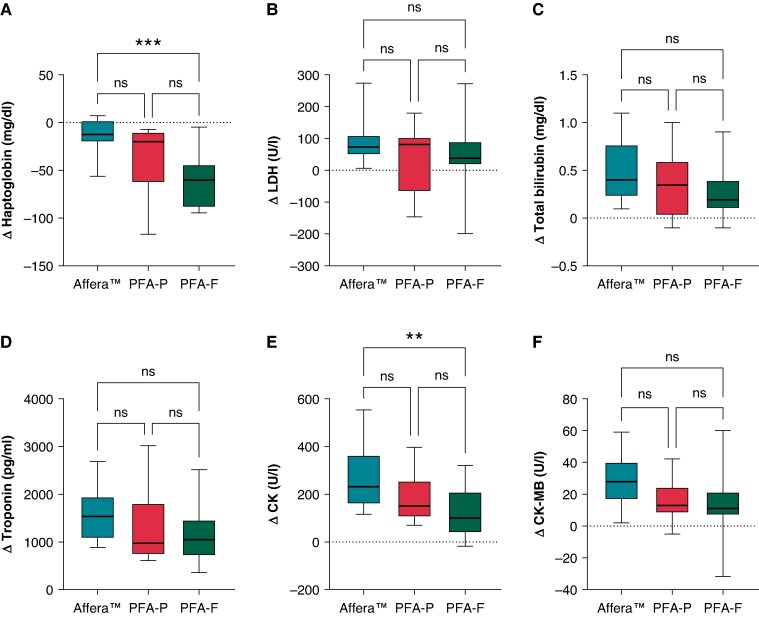
Comparison of haemolysis parameters, (*A*) Δhaptoglobin (mg/dL), (*B*) ΔLDH (U/L), and (*C*) Δtotal bilirubin (mg/dL), and myocardial injury parameters, (*D*) Δtroponin (pg/dL), (*E*) ΔCK (U/L), and (*F*) ΔCK-MB (U/L) post-PVI using different PFA platforms. Comparison of haemolysis and myocardial injury parameters between all three modalities post-PVI compared to baseline showed a significantly higher (*A*) Δhaptoglobin decrease in PFA-F compared to Affera^TM^ and no significant difference in (*B*) ΔLDH and (*C*) Δbilirubin between all three PFA modalities. Myocardial injury markers (*D*) Δtroponin and (*F*) ΔCK-MB were comparable between all three groups. (*E*) ΔCK was significantly higher in Affera^TM^ compared to PFA-F. ns, *P* > 0.05; **, *P* < 0.01; ***, *P* < 0.001.

In comparison, the overall decrease in haptoglobin levels was more pronounced in patients treated with the pentaspline and circular PFA catheter compared to the lattice-tip catheter (Δhaptoglobin: −13.8 ± 18.5 vs.—36.8 ± 35.9 vs. −60.7 ± 26.3 mg/dL, *P* = <0.001, for Affera^TM^, PFA-P, and PFA-F, respectively; *Figure 3*). No significant difference was observed in LDH (ΔLDH: 91.4 ± 69.8 vs. 36 ± 102 vs. 47.5 ± 108 U/L, *P* = 0.316, for Affera^TM^, PFA-P, and PFA-F, respectively) and total bilirubin levels (0.475 ± 0.305 vs. 0.367 ± 0.345 vs. 0.250 ± 0.253 mg/dL, *P* = 0.178, for Affera^TM^, PFA-P, and PFA-F, respectively) between the three PFA modalities (*Figure 3*). No haemolysis-related complications occurred, and creatinine levels remained unchanged post-procedure compared to pre-procedure. Also when comparing Affera™, a monopolar biphasic pulsed field waveform, with both bipolar biphasic PFA waveforms (PFA-P and PFA-F), a consistent pattern was observed: a significantly greater decrease in haptoglobin was noted in the bipolar biphasic PFA group (−13.8 ± 18.5 vs. −50.2 ± 32.5 mg/dL, *P* = 0.001), while no significant increases were seen in LDH (*P* = 0.127) or bilirubin levels (*P* = 0.108).

A trend towards a greater increase in myocardial injury markers (CK, CK-MB, troponin) after ablation using the lattice-tip catheter and a monopolar, biphasic PF waveform can be noted, although the increase of myocardial injury markers is not statistically significant in a head-to-head comparison of the different systems, except for a significant increase in CK levels in the lattice-tip group compared to PFA-F (ΔCK: 232 [168] vs. 102 [144] U/L, *P* = 0.007; *Figure 3*).

**Figure 4:**
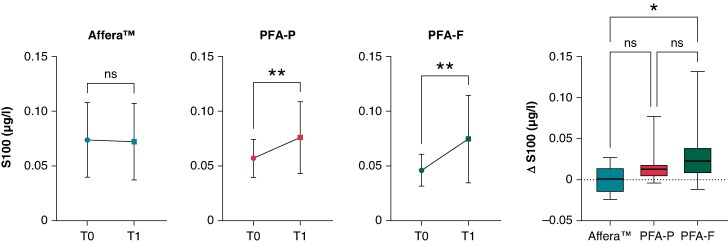
Temporal dynamics of S100 baseline (T0) compared to Day 1 (T1) post-ablation and ΔS100 (ug/L). Analysis of temporal dynamics revealed no significant change in S100 levels in the Affera^TM^ group, whereas a significant post-procedural increase was observed in both PFA-P and PFA-F compared to baseline. Comparison across all three PFA modalities demonstrated a significantly greater ΔS100 increase in PFA-F compared to Affera^TM^. ns, *P* > 0.05; *, *P* < 0.05; **, *P* < 0.01.

Patients treated with biphasic bipolar waveforms using the pentaspline or circular PFA catheter showed an increase in S100 concentration post-procedural (PFA-P: 0.054 [0.023] vs. 0.07 [0.035], *P* = 0.005; PFA-F: 0.048 [0.024] vs. 0.072 [0.047] μg/L, *P* = 0.002), whereas in patients treated with Affera^TM^ and the lattice-tip catheter, no effect on S100 was noted (0.076 [0.035] vs. 0.075 [0.037] μg/L, *P* = 0.854; *Figure 4*). In direct comparison, we observe a significantly higher increase in ΔS100 in PFA-F compared to Affera^TM^ (*P* = 0.020) and a trend towards higher increase in PFA-P vs. Affera^TM^, however not reaching statistical significance (*P* = 0.143; *Figure 4*).

## Discussion

The current study gives important insights into PFA-related haemolysis, the amount of myocardial injury, and the effects on neural injury after AF ablation using different PF platforms. The main results are the following:

1. Post-procedural haemolysis after PFA for the treatment of AF is common and occurs regardless of the PFA waveform, ablation system, and catheter type or shape.
2. The severity of haemolysis appears to be more pronounced for bipolar, biphasic PFA using the pentaspline catheter, with no haemolysis-related complications observed in our cohort. However, given the limited sample size and rare cases of haemolysis-induced renal failure reported in large registries, these findings should be interpreted with caution.
3. There is a trend towards a higher increase in myocardial injury markers after ablation using the lattice-tip catheter and a monopolar, biphasic PF waveform, although the increase of myocardial injury markers is not statistically significant in a direct comparison of different ablation systems.
4. In contrast to bipolar, biphasic PFA ablation using the pentaspline or circular catheter, focal monopolar, biphasic PFA using the lattice-tip catheter induces no neuronal damage quantified by a neuron-specific biomarker.

The pentaspline catheter, as first clinically tested PFA catheter for the treatment of AF, received approval from the European regulatory authorities in 2021. Initial studies indicated that PFA is a rapid and effective technique to achieve PVI.^9,10^ First, direct comparisons with established thermal ablation proved non-inferiority of PFA in terms of acute and long-term efficacy.^11,12^ In addition to its promising efficacy profile, PFA, due to its high cardiomyocyte selectivity, seems to overcome typical (thermal) complications of AF ablation.^13^ However, other new complications seem to arise with this novel energy source. For example, an increased incidence of coronary spasms has been observed during PFA.^14,15^ Furthermore, meanwhile it is known that PFA can be associated with a risk of haemolysis. Of note, in the largest global database of PFA, MANIFEST 17K, which includes over 17 000 patients, several instances of haemolysis were reported, mostly as transient haemoglobinuria without renal injury. Haemolysis-induced renal failure occurred in only 0.03% of patients, indicating that while haemolysis is observed after PFA, progression to clinically relevant complications is rare.^14^ Meanwhile, various ablation systems from different manufacturers are available, which differ in applied PF waveform and the catheter shapes and configuration used.^3^ So far, it is unknown whether all currently available PFA systems bear the same risk of haemolysis.

Our data show that post-procedural haemolysis after PFA for the treatment of AF is common and occurs regardless of the PF waveform and catheter shape or configuration used. In our cohort, haemolysis tended to be more pronounced for bipolar PFA using the pentaspline catheter (*Figure 3*), whereas monopolar lattice-tip PFA was associated with the lowest haemolytic profile, which is in line with previous studies.^16^ Of note, no haemolysis-related complications (e.g. renal failure) were observed in our cohort. However, given the limited sample size and the rare but reported cases of haemolysis-induced renal failure in large registries,^14^ our data should not be interpreted as evidence of clinical non-relevance. Prior studies, predominantly collected from bipolar pentaspline catheter procedures, suggested that the extent of ablation correlates with the risk for haemolysis as well as haemolysis-induced renal failure.^5,17^ Furthermore, it was shown that focal PFA catheters cause less haemolysis per procedure and per application than large-footprint PFA catheters. In our cohort, the number of PFA applications did not correlate with the risk and extent of haemolysis during ablation using the Affera^TM^ mapping and ablation system. One can therefore assume that extensive ablation should be avoided, especially with larger single-shot PFA catheters, to minimize the risk of post-procedural haemolysis. Whether monopolar PFA applications result in less haemolysis than bipolar PFA applications remains unclear and would require a study with similar catheter design but the ability to apply monopolar as well as bipolar PFA applications, which is not available yet.

Interpretation of our findings regarding neural injury via S100 release requires careful consideration of the biomarker used. S100B is not a specific marker for a distinct neural structure but rather reflects neural distress more broadly, being expressed in different glial populations and even in non-neural tissues. Accordingly, elevated S100B levels have been described in various conditions such as acute brain injury, neurodegenerative or psychiatric disorders, and even extracerebral inflammatory diseases.^18^ Nonetheless, evidence indicates that S100B is also expressed and released from cardiac glial cells within the intrinsic cardiac autonomic nervous system during AF ablation.^19,20^ Accordingly, S100B has been applied as a surrogate biomarker for neural injury in several clinical ablation studies. Notably, Tohoku *et al.*^21^ demonstrated significantly lower S100B release after PFA compared to cryoballoon ablation (CB), with differences persisting even after exclusion of patients with silent cerebral ischaemia on MRI. Similarly, Lemoine *et al.*^22^ found that post-PFA S100B levels were significantly lower than after CB, and the ratio of S100B to high-sensitivity troponin I was significantly reduced, indicating comparatively less neural injury per myocardial lesion. However, unlike Tohoku *et al.*, we did not perform systematic post-procedural MRI to exclude silent cerebral ischaemia as a potential source of S100B release and therefore cannot exclude central neuronal injury as an additional cause for S100 release after ablation in our cohort. In our analysis, focal monopolar, biphasic PFA using the lattice-tip catheter showed no neuronal damage quantified by S100 increase. In contrast, bipolar, biphasic PFA ablation using the pentaspline or circular catheter was associated with at least a small but significant effect on neural injury surrogate biomarkers (*Figure 4*). A damage of ganglionated plexi by epicardial bipolar PFA has also been previously demonstrated in pigs.^23^ From a biophysical point of few, the effect of endocardial PFA applications on epicardially located neural structures should be clearly less pronounced. Accordingly, for all PFA systems, the release of S100 post-procedure was negligible in comparison to thermal ablation as previously described.^21,22^ While a small neural damage was detected after bipolar PFA applications, monopolar ablation with the Affera^TM^ system was not associated with any increase in neural biomarkers. Whether this really reflects a group effect or whether this is a result of a point-by-point PFA application by Affera^TM^ vs. a wide ostial and antral PFA application by the bipolar biphasic single-shot devices needs to be studied in further investigations. Besides this, also the clinical relevance of the small but detectable neural damage remains unclear and would require studies focusing on heart rate variability or more detailed autonomic testing, which was not the scope of our study.

Previous studies demonstrated that various biomarkers of myocardial injury and inflammation, including troponin, CK, and creatine kinase MB, as well as C-reactive protein, are elevated in patients undergoing thermal ablation.^24,25^ Of note, the amount and timing of myocardial injury biomarker release may differ between different energy sources (e.g. RF vs. cryoablation) due to their considerable differences in lesion formation. The relationships between these biomarkers after an ablation procedure and clinical outcomes, including early and late arrhythmia recurrences of AF, have been investigated in several studies. A meta-analysis on this topic found that a lower post-procedural elevation of myocardial injury biomarkers and increased CRP levels post-procedure may be predictive factors for AF recurrence.^26^ So far, there is only few data on inflammation parameters and the characteristics of myocardial injury biomarker release after PFA.^27,28^ However, PFA lesions are homogeneous with a preserved tissue architecture and are reported to be associated with less tissue inflammation than thermal ablation in animal models^29,30^ . Therefore, PFA bears the potential of reducing the post-ablation inflammatory reaction, which may be predictive of early AF recurrence and causal for post-procedural pericarditis. Furthermore, in a previous study on this topic, Popa *et al.* could demonstrate that PFA seems to result in higher myocardial injury biomarker release when compared to RF ablation, which is in line with preclinical data.^27,31^ Thus, Popa *et al.*^27^ could not confirm an association with higher myocardial injury biomarker release after PFA and improved clinical outcome within their cohort. However, the highly distinct lesion formation mechanisms of the two ablation modalities should also be considered when interpreting the differences in myocardial injury marker release after ablation, since they may impact biomarker release kinetics and may not necessarily reflect lesion quality.

In our study, a trend towards a greater increase in myocardial injury markers after ablation using the lattice-tip catheter and a monopolar, biphasic PF waveform can be noted, although the increase of myocardial injury markers was not statistically significant in a direct comparison of different ablation systems. However, this finding may underline the hypothesis that monopolar PFA results in deeper lesion as recently suggested.^32^ Whether this directly translates into improved acute and long-term lesion quality and whether this therefore is associated with increased arrhythmia-free survival rates after ablation needs to be further investigated.

### Limitations

The findings of our study add important knowledge on haemolysis profile as well as the extent of myocardial and neural injury after PFA ablation using different PFA platforms. However, due to the novelty of the novel mapping and ablation system analysed within this study (Affera^TM^, Medtronic Inc.), sample size is limited. Therefore, and as clinical follow-up is pending so far, the results of our study remain hypothesis generating.

Moreover, the absence of systematic post-procedural cerebral MRI scans to rule out silent cerebral ischaemia as a potential contributor to S100 release, together with the lack of functional autonomic testing such as heart rate variability, further limits the interpretation of our findings.

## Conclusions

Post-procedural haemolysis after PFA for AF treatment is common and occurs regardless of the PFA platform and catheter shapes used. Pulsed field ablation using Affera^TM^ seems to be associated with a high increase in myocardial injury markers when compared to other PFA platforms using bipolar, biphasic PF waveforms. In general, PFA induces at least minimal neuronal damage quantified by neuron-specific biomarkers.

## Data Availability

The data underlying this study are available from the corresponding author upon reasonable request.
