# Correction to: Haemolysis and myocardial and neural injury after monopolar pulsed field ablation using a novel lattice-tip catheter to treat atrial fibrillation

**DOI:** 10.1093/europace/euag187

**Published:** 2026-07-23

This is a correction to: Christian Gold, Paul Pratz, Anastasia Falagkari, Victoria Johnson, Florian Post, Esther Roth, Jana Kupusovic, Julia W Erath-Honold, Dominik Linz, David Leistner, Reza Wakili, Laura Rottner, Haemolysis and myocardial and neural injury after monopolar pulsed field ablation using a novel lattice-tip catheter to treat atrial fibrillation, *EP Europace*, Volume 27, Issue 9, September 2025, euaf210, https://doi.org/10.1093/europace/euaf210

In the originally published version of this manuscript, the Affera system was erroneously described as “monopolar monophasic”. This has been corrected to read “monopolar biphasic” throughout the manuscript.

In the Results and Discussion sections, the term “bipolar” has been added for the comparator systems (PFA-P and PFA-F) to clearly distinguish them from the monopolar biphasic Affera system.

In the Introduction, the word ‘ablation’ was omitted from a sentence. The sentence has been corrected to read: “In addition to its promising efficacy and clinical outcome profile, PFA—due to its cardioselectivity—seems to overcome typical complications of atrial fibrillation (AF) ablation (e.g. atrio-oesophageal fistula or phrenic nerve palsy)” instead of: “In addition to its promising efficacy and clinical outcome profile, PFA—due to its cardioselectivity—seems to overcome typical complications of atrial fibrillation (AF) (e.g. atrio-oesophageal fistula or phrenic nerve palsy)”.

Additionally, an asterisk was inadvertently omitted from the Figure 2 legend. This has been corrected to read “[…] ****, P ≤ 0.0001”.

